# Assessment of tetracycline contamination in surface and groundwater resources proximal to animal farming houses in Tehran, Iran

**DOI:** 10.1186/s40201-016-0245-z

**Published:** 2016-02-01

**Authors:** Allahbakhsh Javid, Alireza Mesdaghinia, Simin Nasseri, Amir Hossein Mahvi, Mahmood Alimohammadi, Hamed Gharibi

**Affiliations:** School of Public Health, Shahroud University of Medical Sciences, Shahroud, Iran; Department of Environmental Health Engineering, School of public Health, Tehran University of Medical Sciences, Tehran, Iran; Center for Water Quality Research, Institute for Environmental Research, Tehran University of Medical Sciences, Tehran, Iran; Center for Solid Waste Research, Institute for Environmental Research, Tehran University of Medical Sciences, Tehran, Iran

**Keywords:** Tetracycline, Groundwater resources, Surface water resources, Drinking water, Animal farms

## Abstract

**Background:**

Antibiotics have been increasingly used for veterinary and medical purposes. The overuse of these compounds for these purposes can pollute the environment, water resources in particular. Tetracycline, among other forms of antibiotics, is one of the most applied antibiotic in aquaculture and veterinary medicine. The present study aimed to tack the traces of tetracycline in the effluents of municipal and hospital wastewater treatment plants, surface and groundwater resources and finally the drinking water provided from these water resources.

**Methods:**

The samples were taken from Fasha-Foyeh Dam, wells located at Varamin Plain, and Yaftabad; and also, wastewater samples were collected from the wastewater treatment plant effluents of Emam Khomeini Hospital and a municipal wastewater treatment plant which its effluent is being released to the surface water of the area covered in this work. 24 samples were collected in total during July 2012 to December 2012. The prepared samples were analyzed using high-performance liquid chromatography.

**Results:**

Based on the results, mean tetracycline levels in surface and ground water at nearby of animal farms was found to vary from 5.4 to 8.1 ng L^-1^. Furthermore, the maximum TC concentration of 9.3 ng L^-1^ was found to be at Yaft-Abad sampling station. Although tetracycline traces could not be detected in any investigated Hospital WWTP effluents, it was tracked in MWWTP effluent samples, in the concentration range of 280 to 540 ng l^−1^.

**Conclusion:**

The results showed that the concentration of TC in water resource near the animal farms is higher than the other sampling stations. This is related to the usage of antibiotic for animals. In fact, it caused the contamination of water resources and could contribute to radical changes in the ecology of these regions.

## Background

Antibiotics have been increasingly used for veterinary and medical purposes. The increase in the use of these compounds affects both the environment and human health; in other words, the active forms of the antibiotics are being excreted from the body via urine and/or feces into the environment. Considering this, the overuse of these compounds can pollute the water resources [1]. It should be noted that there are various pathways in which these compounds enter into both surface and groundwater resources, including run-off, leakage from lagoons, leaching of manure applied to fields, and leaching from animal housing areas [Watanabe et al.]. The presence of antibiotics, TC in particular, in water and soil can cause some allergies and toxicity, since these compounds are still active [2]. For instance, excreted antibiotics in the environment affect almost all the bacterial species forcing them to develop a resistance toward these compounds [3]. Based on the reports of previously conducted studies, the residues of various forms of antibiotics have been detected in the samples taken from surface and groundwater resources and also drinking water [4, 5]. Furthermore, the antibiotics have also been found in the samples taken from the effluents of both municipal and hospital wastewater treatment plant [6, 7].

Tetracycline (TC), among other forms of antibiotics, is one of the most applied antibiotics in aquaculture and veterinary medicine [8]. It should be noted that this antibiotic has been applied in livestock and poultry productions more than the aquaculture medicine. In addition, tetracycline is being discharged into the environment, water resources in particular, through wastewater effluent of drug manufacturing companies, disposal of non-consumable compounds and expired drugs containing tetracycline, and also from animal and agricultural wastes [9, 10]. TC has been classified among the antibiotics frequently detected in sewage, domestic wastewaters, surface and groundwater resources, drinking water, and sludge [11]. Considering the increase in the usage of TC and also the inefficiency of most conventional wastewater treatment processes in removing this antibiotic, the surface and ground water resources are now at more risk of being polluted with TC. Furthermore, it should be noted that there is not any regulation for routine sampling and analyzing TC level in the water resources. Previously conducted studies showed that one of the most widely used antibiotic in animals is tetracycline [8].

**Fig. 1:**
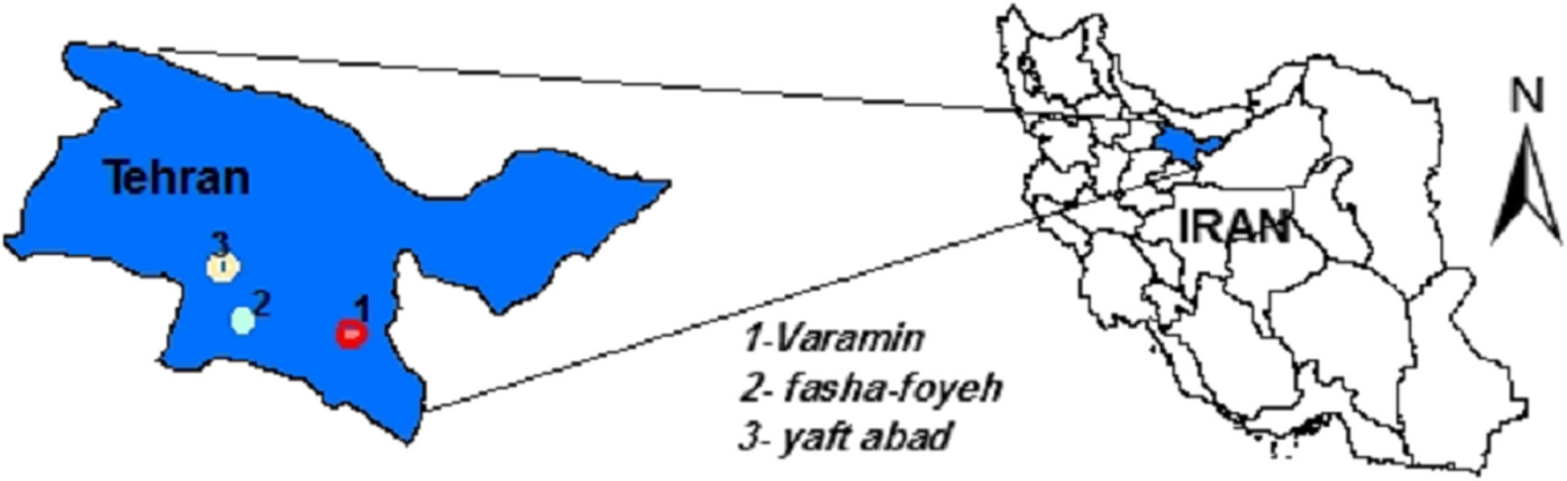
Geographical position of study area and sampling points

This study aimed to tack the traces of tetracycline in a specific route; in other words, tetracycline was strived to detect in the samples taken from the effluents of municipal and hospital wastewater treatment plants, surface and groundwater resources and finally the drinking water provided from these water resources. In this work, TC was selected due to its wide usage and persistence in the environment. Figure 1 shows the sampling locations. It should be noted that these locations are related to each other. In fact, the dam represents the surface water resource receiving the effluents of selected municipal and hospital wastewater treatment plants. The wells, in addition, are located in the vicinity of the main animal farming houses in Tehran, Iran and also the dam. Furthermore, drinking water prepared from using the water resources was also studied.

## Methods

### Sampling stations

In the present work, four samples over 6 months from July 2012 to December 2012 were taken; in total, 24 samples were collected and analyzed in this study. Water samples were taken from tap water, Fasha-Foyeh Dam and also specific wells located at Varamin Plain and Yaftabad. In addition, wastewater samples were collected from the effluents of Emam Khomeini Hospital wastewater treatment plant and a municipal wastewater treatment plant. Figure 1 shows Geographical position of places which the samples were taken.

In this study, two locations were selected to take sample from, namely, Varamin Plain and Yaftabad (i. e. wells nearby animal farming houses) to determine whether TC leached to groundwater resources. In addition, Fasha-Foyeh Dam was selected as surface water resource nearby animal farming houses to assess whether TC has been transported by run-off into the most nearby surface water resource; In other words, the sampling took place at nearby water resources of animal farms which their wastewater effluents are being discharged into the resources. Furthermore, the effluents of municipal and hospital wastewater plants which are being released into the same water resources were considered and analyzed in order to detect TC. The wastewater samples were also taken directly from the effluents of WWTPs to assess the amount of TC released from human sources and compare it with water samples mentioned above. Another spot was also selected (i. e. tap water) to determine whether the drinking water is polluted.

### Preparing and analyzing the samples

The method of analyzing the samples is based on a method applied and proposed in a study by A Pena et al., [3]. At first, samples were allowed to settle at 4 °C for 12–24 h in the dark. Careful handling prevented re-suspension of settled particles, and the supernatant of the samples was used for analysis instead of a filtered aliquot [12]. Since antibiotics are present in surface and ground waters and other resources at trace levels, pre-conditioning by suitable sorbent is a necessary step in sample preparation. The average recoveries 80 ± 3 were obtained using strata C18-E cartridge. Detection frequency of tetracycline in water sources and WWTP effluent is shown in Table 1.

**Table 1:** HPLC gradient program used for determination of TC

Time (min)	Mobile phase A (%)	Mobile phase B (%)
0	10	90
7	40	60
7.5	50	50
12.95	10	90
13		End

According to a study conducted by Shalaby et al., the strata C18-E cartridge was applied to buffer extract the residue; after sample loading, the cartridge was washed by 10 ml of 5 % methanol mixed in water, and tetracycline was eluted with a mixture of 10 ml of methanol and 0.01 M oxalic acid [13].

The samples were taken by syringe and filtered through 0.45 μm membrane at pre-selected time intervals; then, the samples were measured using high-performance liquid chromatography (HPLC). HPLC consisted of a Knauer LPG pump, an EZ-chrom HPLC system manager program and a UV detector (k-2500). The UV–detector was set at the maximum absorption wavelength of 365 nm. Aliquots of 100 μL were injected manually using a model SGE injection valve (SGE. Australia). MZ-analysentechnik ODS-3 C18 (4.6 mm × 250 mm) packed with 5 μm spherical particles was used for separation. An Acetonitrile (A) aqueous oxalic acid 0.01 M (B) mixture was used as mobile phase at 30^0^C temperature with a constant flow rate of 1.0 mLmin^−1^. The used mobile phase are shown in Table 1 [3].

## Results and discussion

This work is the first survey of the contamination of surface and ground water resources with TC in Tehran, Iran. The results showed that the mean concentration of TC in water resources, both surface and groundwater, varies from 5.4 to 8.1 ng L^-1^among which Yaft-abad station with 9.3 ng L^-1^ level of TC was found to have the maximum TC concentration; The results are shown in Table 2. The concentration of TC in the samples taken from surface water resource (i. e. Fasha-foyeh- (dam)), was found in the range of 5.7 to 8.7 ng/L. Detected levels of this antibiotic in surface water resources could be explained by the fact that tetracycline of veterinary wastes finds its way into the surface and ground water resources. Run-off is one of the main ways in which antibiotics leach into particularly surface water resources [14, 15]. Considering the location of animal farming houses in vicinity of Fasha-foyeh- (dam), this could be a probable cause of finding trace amount of TC in the taken samples from this location. There is also another source of polluting the surface water resources, as taken into account in this study, and it is the release of wastewater effluents into the resources [15]. The concentration of TC in MWWTP effluent samples ranged from 280 to 540 ng l^−1^. Considering the fact that most of the antibiotics have high affinity to soil compounds [16, 17], tetracycline in particular, the main source of TC in the surface water resource studied in this work could be the effluent of MWWTP. This result is in line with the results of previously conducted studies on the presence of antibiotics in the water resources [1, 13]. In addition, the concentration of TC was negligible in the samples taken from the effluents of Hospital wastewater treatment plant. The main reason is that TC is no longer being used among hospitalized patients. In a study conducted in the United States of America, the concentration of tetracycline in the effluent of MWWTP was reported to be between 170 and 850 ng L^-1^ [18]. In a study conducted by Miao et al., 150 to 970 ngL^-1^ concentration of TC was reported in Canada [19]. Comparing with these findings, the concentrations of TC detected in the samples taken from WWTPs effluents in this study are at lower levels.

**Table 2:** Mean concentrations of tetracycline in water resource and wastewater treatment plant effluent

Location	TC concentration (ngL^-1^)	
Yaft-abad – (well)	Mean	8.1
	Max	9.3
	Min	6.9
	*n* = 4	
Fasha-foyeh- (dam)	Mean	6.4
	Max	8.7
	Min	5.7
	*n* = 4	
Varamin- (well)	Mean	5.4
	Max	7.2
	Min	4.4
	*n* = 4	
Tap water	ND	
	*n* = 4	
HWWTP effluent	ND	
	*n* = 4	
MWWTP effluent	Mean	540
	Max	630
	Min	280
	*n* = 4	

*ND* non-detectable

As mentioned above, the highest concentration of TC was found in the samples taken from ground water resources. Although TC has high affinity to soil compounds, the presence of this antibiotic in the samples even in this range is of high importance. It should be noted that the overuse of these drugs causes them to be found in the water resources due mainly to the saturation of soil capacity; in fact, this phenomenon is known as terracumulation [20, 21]. When the soil becomes saturated with TC, the infiltration of water originated from rain into groundwater resources can carry this antibiotic contributing to the pollution of these resources [22]. Considering the fact that TC is still an active antibiotic when it is bounded to soil particles, the presence of it in groundwater resource is indeed a major problem.

In addition, four samples were taken from tap water to determine whether any concentration of TC is in drinking water. The water treated and distributed among the people of the region we took samples from came from a water treatment plant which uses the water resources (i. e. surface and groundwater) mentioned above. As shown in Table 2, the concentration of TC in these samples was negligible.

## Conclusion

This work was aimed to survey the contamination of surface and ground water resources with TC in Tehran, Iran. Based on the results, the water sources studied in this work are contaminated with TC and the presence of other types of antibiotics is also probable. The release of TC from the animal farming areas can be implied to be the main source of groundwater resources pollution with this antibiotic. In addition, the release wastewater effluent from WWTP can be considered as a potential source for the contamination of surface water resource with TC. In fact, the presence of this antibiotic in the environment can cause the mutation of the bacterial species and make them resistant to the antibiotics; and also, negatively affect those who use the water from this resource. In this regard, regular monitoring of the presence of antibiotics mostly used in these areas in order to prevent further damage to the environment. In conclusion, so we recommend more monitoring of the existence of antibiotics residues in water resource near the animal farms.

## Abbreviations

TC: tetracycline
HPLC: high-performance liquid chromatography
WWTP: wastewater treatment plant
MWWTP: municipal wastewater treatment plant
